# Rectal Carriage of Extended-Spectrum Beta-Lactamase-Producing Gram-Negative Bacilli in Community Settings in Madagascar

**DOI:** 10.1371/journal.pone.0022738

**Published:** 2011-07-29

**Authors:** Perlinot Herindrainy, Frédérique Randrianirina, Rila Ratovoson, Elisoa Ratsima Hariniana, Yves Buisson, Nathalie Genel, Dominique Decré, Guillaume Arlet, Antoine Talarmin, Vincent Richard

**Affiliations:** 1 Institut de la Francophonie pour la Médecine Tropicale, Ventiane, Laos; 2 Institut Pasteur de Madagascar, Antananarivo, Madagascar; 3 Laboratoire de Bactériologie, Faculté de Médecine Pierre et Marie Curie, UPMC, Paris France; Institut Pasteur, France

## Abstract

**Background:**

Extended-spectrum ß-lactamase-producing Enterobacteria (ESBL-PE) emerged at the end of the 1980s, causing nosocomial outbreaks and/or hyperendemic situations in hospitals and long-term care facilities. In recent years, community-acquired infections due to ESBL-PE have spread worldwide, especially across developing countries including Madagascar.

**Objectives:**

This study aimed to determine the prevalence and risk factors of intestinal carriage of ESBL-PE in the community of Antananarivo.

**Methods:**

Non-hospitalized patients were recruited in three health centers in different socio economic settings. Fresh stool collected were immediately plated on Drigalski agar containing 3 mg/liter of ceftriaxone. Gram-negative bacilli species were identified and ESBL production was tested by a double disk diffusion (cefotaxime and ceftazidime +/− clavulanate) assay. Characterization of ESBLs were perfomed by PCR and direct sequencing . Molecular epidemiology was analysed by Rep-PCR and ERIC-PCR.

**Results:**

484 patients were screened (sex ratio  = 1.03, median age 28 years). 53 ESBL-PE were isolated from 49 patients (carrier rate 10.1%). The isolates included *Escherichia coli* (31), *Klebsiella pneumoniae* (14), *Enterobacter cloacae* (3), *Citrobacter freundii* (3), *Kluyvera spp.* (1) and Pantoae sp.(1). In multivariate analysis, only the socioeconomic status of the head of household was independently associated with ESBL-PE carriage, poverty being the predominant risk factor.

**Conclusions:**

The prevalence of carriage of ESBL in the community of Antananarivo is one of the highest reported worldwide. This alarming spread of resistance genes should be stopped urgently by improving hygiene and streamlining the distribution and consumption of antibiotics.

## Introduction

New classes of enzymes conferring resistance to β-lactam antibiotics have emerged over the last few decades, due to antibiotic selection pressure; most alarming are the extended spectrum β-lactamases (ESBLs) produced by enteric pathogens that have spread worldwide since their first description in 1983 [1]. Typically, ESBLs hydrolyze third generation cephalosporins and aztreonam, but not carbapenems, and are inhibited by clavulanic acid and tazobactam [2]. Many ESBL-producing pathogens also express plasmid-encoded multidrug resistance. Consequently, effective antibiotic therapy for treating these infections is limited to a small number of expensive drugs. More than 200 types of ESBL, the results of mutations, have been described in various species of the Enterobacteriaceae family and other non enteric organisms, including *Pseudomonas aeruginosa* and *Acinetobacter sp*. TEM- and SHV-type β- lactamases, mainly produced by *Klebsiella pneumoniae*, have spread throughout hospital settings, and CTX-M enzymes, mainly produced by *Escherichia coli*, have become predominant in the community [3], [4].

Over recent years, there has been increasing recognition of the importance of community-acquired infections due to ESBL-producing *E. coli*. ESBLs were initially associated with nosocomial outbreaks caused by single enzyme-producing strains, but recent studies have revealed the existence of more complex situations, with significant increases in the frequency of community isolates [5], [6]. Fecal carriage of ESBL-producing isolates is now widely studied in hospitals but few studies have evaluated intestinal carriage in populations in the community. Surveys since 2000 in several European countries and Canada have shown an alarming trend of associated resistance to other classes of antimicrobial agents among ESBL-producing organisms isolated from community sites [1], [3], [7]. These phenomena are contributing to changes in the epidemiology of antibiotic resistance. The high antimicrobial drug resistance rates observed in low-resource countries are probably due to a combination of several factors, including irrational antimicrobial drug use and poor conditions of sanitation, both of which are believed to play major roles [8].

ESBL-producing enterobacteria (ESBL-PE) were first isolated in Madagascar between 2005 and 2006 from community-acquired urinary tract infections [9]. They were subsequently isolated during an epidemic affecting two pediatric units in 2006. More recently, ESBL-PE have been isolated from several infections acquired in various surgical and intensive care units of Antananarivo [10] and from patients hospitalized in a pediatric department of a large teaching hospital in Antananarivo [11].

The objectives of this study were to investigate the prevalence and predisposing factors of intestinal carriage of ESBL-PE in the community.

## Results

### Prevalence of ESBL-PE carriage

Of the 515 patients questioned, 484 were enrolled. The sex ratio (male/female) was 1.03 and the median age was 28 years (Inter Quartile Range - IQR: 9–40 yrs). Three age groups were distinguished: 0–4 years (12.8%), 5–14 years (23.8%), 15 years and more (63.4%). Most heads of household had at least a high school level of occupation (45.1% university level and 40.8% secondary school level); furthermore, in view of their occupation, 46.7% were unskilled or semi-skilled workers and 4.7% were professionally inactive.

A total of 49 patients were found to be ESBL-PE carriers. Four subjects harbored two different strains, bringing the total number of ESBL-PE isolates to 53. There was no significant difference between sex or age groups (Table 1).

**Table 1:** Characteristics of patients and analysis of the risk factors.

	Total		ESBL +		ESBL −			
	(n = 484)		(n = 49)		(n = 435)			
	N	(%)	N	(%)	N	(%)	OR	p-value
**Sex**								
Male	245	(50.7)	23	(46.9)	222	(51.2)	0.84	0.57
Female	238	(49.3)	26	(53.1)	212	(48.8)	–	
**Age group (years)**								
<5	62	(12.8)	6	(12.3)	56	(12.9)	–	0.61
5 to 14	115	(23.8)	10	(20.4)	105	(24.2)	0.93	
≥15	306	(63.4)	33	(67.3)	273	(62.9)	1.15	
**Median age** [IQR]	28	[9-40]	25	[9-37]	28.5	[9-40]		0.72
**Head Family Occupation**								
Without occupation	23	(4.7)	4	(8.2)	19	(4.4)	–	**0.02**
Unskilled	116	(23.9)	13	(26.5)	103	(23.7)	0.6	
Semi skilled	110	(22.7)	15	(30.6)	95	(21.8)	0.5	
Non-manual employee	147	(30.4)	15	(30.6)	132	(30.3)	0.7	
Employers and managers	88	(18.2)	2	(4.1)	86	(19.8)	0.1	
**School level**								
No schooling	8	(1.6)	0	-	8	(1.8)	–	0.14
Primary schooling	60	(12.4)	10	(20.4)	50	(11.6)	–	
Secondary schooling	197	(40.8)	22	(44.9)	175	(40.3)	0.7	
High schooling	218	(45.1)	17	(34.7)	201	(46.3)	0.5	
**Household members**median [IQR]	4	[4]–[6]	4	[4]–[5]	5	[4]–[6]		0.36
**Number of rooms**median [IQR]	3	[2]–[4]	2	[1]–[3]	3	[2]–[4]		**0.01**
**Ratio inhabitant/room**	1.5	[1]–[3]	2	[1]–[4]	1.5	[1]–[3]		**0.04**
**Television** *yes*	427	(88.2)	42	(85.7)	385	(88.5)	0.77	0.57
**Mobile phone** *yes*	425	(87.8)	39	(79.6)	386	(88.7)	0.49	0.08
**House characteristics**								
Individual house	222	(45.9)	27	(55.1)	195	(44.8)	1.51	0.17
Flat	262	(54.1)	22	(44.9)	240	(55.2)	-	
**Shower unit** *yes*	419	(86.6)	41	(83.6)	378	(86.9)	0.77	0.54
**Past history**(From 3 to 12 months)								
** antibiotherapy**	156	(32.8)	20	(12.3)	136	(31.2)	1.51	0.18

Occupation of the head of family had a significant effect on BLSE-PE carriage: prevalence was lower (2.3%) in the manager than unemployed (17.4%) group. Carriage also differed according to the number of rooms in the residence: the houses of ESBL-PE carriers (median number of rooms: 2 rooms - IQR: [1]–[3]) were smaller than those of non-carriers (median number of rooms: 3 rooms - IQR: [2]–[4]) (Table 1).

### Characteristics of ESBL-PE isolates

The 53 ESBL-PE isolates recovered during the study were *E. coli* (n = 31), *K. pneumoniae* (n = 14), *Enterobacter cloacae* (n = 3), *Citrobacter freundii* (n = 3), *Kluyvera spp*. (n = 1), and *Pantoa spp*.(n = 1).

All isolates were susceptible to amikacin and imipenem, and most were resistant to co-trimoxazole. The main associated resistances were observed in *E. coli* and *K. pneumoniae*, and were against gentamicin, tobramycin and fluoroquinolones (Table 2).

**Table 2:** Antimicrobial resistance of ESBL-producing isolates (number of tested isolates in brackets).

Drugs	*E. coli*	*K.Pneumoniae*	*E.cloacae*	*C freundii*
	% (n = 31)	% (n = 14)	% (n = 3)	% (n = 3)
*Amoxicillin*	100.0	100.0	100.0	100.0
*Ticarcillin*	100.0	100.0	100.0	100.0
*Ceftazidime*	100.0	92.8	100.0	100.0
*Gentamicin*	54.8	57.1	66.7	100.0
*Tobramycine*	73.3	69.3	100.0	100.0
*Imipenem*	0.0	0.0	0.0	0.0
*Amikacin*	0.0	0.0	0.0	0.0
*Nalidixic acid*	74.2	64.3	66.6	33.3
*Ciprofloxacin*	71.0	57.1	0.0	33.3
*Co-trimoxazole*	83.9	100.0	100.0	100.0

As expected the most common CTX-M types were CTX-M-15 (n = 46) Others enzyme were SHV-2a in one *K. pneumoniae*, SHV-12 in 2 E. cloacae, CTX-M-1 in one *E. coli* et CTX-M-3 in 2 *E. coli*. . The chromosomal *bla* gene of the *Kluyvera spp* has not been characterized.

Molecular characterization of the *E. coli* strains showed that they belonged to 6 different phylogenetic groups. The A1 group was the most frequent (14 of 31 strains, 45%). Molecular analysis evidenced 3 clones among *E. coli* strains in the A1 group; 2 clones with 3 strains and one clone with 2 strains. No clone could be evidenced among *K. pneumoniae* or *E. cloacae*.

### Risk factors for ESBL-PE carriage

Univariate analysis identified only three variables as being associated with ESBL-PE carriage (Table 1): occupation of the head of household (p-value = 0.02), number of rooms occupied by the family (p-value = 0.01) and ratio inhabitant per room (p-value = 0.04). We also introduced into the initial logistic regressive model variables with p-value<0.20: educational level of the head of family, ownership of a mobile phone, and history of antibiotherapy (Table 1). Multivariate analysis confirmed the occupation of the head of household as being independently linked to ESBL-PE carriage: indeed, the probability of being an ESBL-PE carrier was significantly higher in the “Unemployed” (OR: 9.1, CI95 [1.6-53.9]), “Unskilled” (OR: 5.4, CI95 [1.2–24.8]) “Semi skilled” (OR: 6.7, CI95 [1.5–30.4]) and “Non-manual employees” (OR: 4.7, CI95 [1.1–21.3]) groups than among managers.

## Discussion

This study aimed to estimate the prevalence of intestinal carriage of ESBL-PE in the population of Antananarivo and to investigate its determinants. We found the prevalence to be 10.1%, one of the highest reported worldwide and much higher than the 1.5% reported in Brazil [17], 2.4% in Lebanon [18] and 3.7% in Spain [19]. It is more similar to the 7% carriage rate for ESBL-producing *E. coli* observed in China among elderly [20] and the 8% carriage rate of ESBL-PE on admission to hospital in Israel [21]. CTX-M-15 was as expected the most prevalent ESBL among all species. Our results confirm those of a previous study performed in Madagascar [10]. During the 1990s, CTX-M enzyme, especially CTX-M-15 emerged worldwide in the community, mostly detected in *E. coli* from urinary tract infections. These enzymes have spread widely through various enterobacteria, by the transmission of plasmids and mobile genetic elements rather than by clonal dissemination of a bacterial species. The progenitors of CTX-M have been identified on the chromosome of *Kluyvera spp* which are non-pathogenic environmental enterobacteria [22]. Interestingly, one of the 53 ESBL-PE isolated in our study belonged to the genus *Kluyvera*.

This is the first survey conducted in Madagascar on the intestinal carriage of ESBL-PE in the community. The emergence of ESBL-producing *E. coli* strains in community-acquired urinary tract infections has been described previously in Antananarivo [9]. More recently, a study on admissions to a pediatric hospital in Antananarivo revealed a prevalence of rectal carriage of ESBL-PE as high as 22.1% [11]. Our study, conducted on a sample of people who had not consumed any antibiotics for at least three months, confirms the alarming spread of the carriage of multiresistant bacteria through the community in Madagascar.

The main risk factors for ESBL-PE infection in non-hospitalized patients have been identified by a multinational survey: recent antibiotic use, residence in a long-term care facility, recent hospitalization, age 65 years, and male gender [23]. Interestingly in our study, the prevalence of rectal carriage of ESBL-PE in the community was not related to age or sex, but was significantly dependent on socio-economic status: poverty was the main risk factor. Predisposition to ESBL-PE carriage in households where the head is unemployed or has unskilled work has been reported in Israel [24]. The highest carrier rates were observed for people with low or intermediate income and this suggests that poor sanitary conditions favor the transmission of ESBL-PE by the fecal-oral route. However, our study failed to demonstrate significantly associated factors involving equipment or hygiene practices. The fact that the prevalence of carriage did not increase with age suggests that it is evenly spread throughout the disadvantaged community of Antananarivo, resulting from continuous exchanges from person to person mediated by drinking water, food and dirty hands.

The molecular typing of the strains evidenced a great variety of strains. However, 3 clones were detected with 3 strains in two clones and 2 strains in one clone. Curiously, these 3 clones which produce the CTX-M-15 ESBL belong to the phylogenetic group A1 (a commensal and less virulent group) whereas the predominant clone (producing the same enzyme) found in infections, worldwide is the *E. coli* O25b-ST131. Data concerning the patient failed to evidence any familial link between the patients and the addresses were different. However, for one clone the three patients lived at Isotry in low-income neighborhoods, for the second two patients were from high income people attending Institut Pasteur de Madagascar and the third lived in the low income neighborhoods at Isotry. For the third clone, the two patients attended a care center on private practice. The diffusion of CTX-M producer clones in the community has been previously described in Cambodia [14]. These clones in patients from different locations may reflect the mediocre hygiene, the dissemination of enterobacteria through the environment or a contamination of the patients at the same source through food. This way of contamination has previously been suggested [14], [25].

The predominance of *E. coli* among the ESBL-PE isolated confirms the results of a previous study with children on admission to a pediatric ward [11]. This pathogen is the commonest Gram-negative bacterial species, widespread in the community and in healthcare settings other than hospitals; *K. pneumoniae* is more often isolated from nosocomial infections.

The diversity of ESBL-PE reflects interspecies dissemination of resistance genes in the community, a phenomenon favored by selection pressure from antibiotics. Self-medication and the availability of antimicrobials, including amoxicillin and cotrimoxazole, without medical prescription from street vendors remain a concern in Madagascar [9].

Most ESBL-PE carriers in the community are not at risk of infection but are potential sources for human to human transmission [26]. Gut colonization by a strain of ESBL-PE may last several weeks, months or years, depending on possible recontamination and antibiotic pressure. Community-onset infections with ESBL-PE are increasingly reported and require an appropriate response, particularly as concerns empirical antibiotic therapy for Gram-negative infections.

In practice, the frequency of associated resistances to beta-lactams, aminoglycosides and fluoroquinolones limits the therapeutic choice to carbapenems and amikacin. In Madagascar, these antibiotics are not available because they are too expensive.

Poor hygiene and uncontrolled use of broad-spectrum antibiotics are the main determinants of high prevalence of carriage of ESBL in the Malagasy community. It is feared that these trends will worsen, especially among the poorest classes, and that infections intractable to available treatment are increasingly frequent. The spread of multidrug-resistant pathogens has become a major concern in low-income countries. Prevention strategies based on a strict policy of antibiotic use are urgently required.

## Methods

### Setting

This study was conducted in Antananarivo (*Commune Urbaine d'Antananarivo or CUA*), the capital city of Madagascar, located in the central highlands. According to the civic authorities, CUA had a population of about 1.5 million in 2004. Antananarivo consists of administrative, commercial, industrial and residential areas, with patches of agricultural land that are mostly rice fields. The city is divided into six administrative districts (*Firaisana*).

### Study population

The recruits were patients of all ages attending for the first time one of three health centers in poor (Isotry), moderate (Ivato) and comfortable (clinical biology laboratory of the Institut Pasteur or care centre in private practice) areas of the city. They were also enrolled before treatment. Individuals with a current history of infection or fever syndrome or a history of hospitalization within the past 12 months or antibiotic therapy within three months were excluded from the study. If several members of the same household could be included, just one was selected by randomization. The sample size was calculated on the basis of an estimate of the prevalence of ESBL-PE carriage in the population of between 10% and 20%: the recruitment of 150 subjects at each site allowed an accuracy of 5 to 7%, with alpha risk 5% and power 80%.

### Data Collection

Epidemiological data were collected from February 2009 to June 2009 through questionnaires and included socioeconomic conditions (age, gender, educational level etc.) and information about the number of siblings, type of residence, water supply and area of residence.

The different occupation types has been classified in five groups: “Employers and managers”, “non-manual employee” (secretarial occupations, administrative assistant…), “semi skilled employee” (manual employee with limited skill as chauffer, mechanic…), “unskilled employee” (manual employee with no skill) and “without occupation”.

### Laboratory methods

Fresh stools collected from consenting subjects were immediately plated onto Drigalski agar supplemented with 3 mg/liter of ceftriaxone. The plates were forwarded to the Institute Pasteur of Madagascar within 4 hours and incubated for 24 to 48 hours at 37°C. Every colony type was tested for Gram staining and oxidase activity. Oxidase-negative Gram-negative bacilli were identified with the API 20E system (bioMérieux, Marcy l'Etoile, France). Other bacteria were kept in skimmed milk at −20°C for further analysis. The resistance phenotype and the double-disk synergy test with conventional combinations (cefotaxime, ceftazidime, ceftriaxone vs clavulanic acid) [12] were used to assess ESBL production. For strains of *Enterobacter cloacae*, the double disk potentiation test was performed with clavulanic acid and cefepime. Confirmatory tests were performed according to the Clinical and Laboratory Standards Institute's recommendations (CLSI-2005).

Antibiotic susceptibility testing by a disk diffusion method on Mueller-Hinton agar was used according to the recommendations of the Antibiogram Committee of the French Microbiology Society (ACFMS) for the characterization of ESBLs [3]. In addition, susceptibility to each amoxicillin, ticarcillin, gentamicin, tobramycin, amikacin, imipenem, nalidixic acid, ciprofloxacin, and cotrimoxazol was tested. The OSIRIS system (Biorad, Marne la Coquette, France) was used to measure the diameters of inhibition.

The ESBLs were characterized by direct sequencing of PCR products obtained with specific primers of TEM, SHV and CTX-M–type beta-lactamase as previously described [13], [14].

The molecular epidemiology of all strains (except the *Pantoea sp*. and the *Kluyvera sp*. strains) has been done by Rep-PCR and ERIC-PCR as previously described [13].

The phylogenetic group of *E. coli* has been characterized as previously described [14] and the characterization of the virulent clone of *E. coli* O25b-ST131 has been determined by the method of Clermont et al. [15].

### Statistical analysis

Analyses were performed with R Software [16] on three levels: descriptive, univariate and multivariate.

Means and standard deviations (SD) were calculated for quantitative variables and proportions for categorical variables. The Chi–square test and Fisher's exact test were used for univariate analysis, with ANOVA and Kruskall Wallis tests for comparison of medians. P-values <0.05 were considered to be statistically significant.

Explanatory variables associated with a p-value less than 0.20 were analyzed by logistic regression to investigate the confounding factors.

A multivariate logistic regression model was employed with stepwise backward elimination of non-significant variables, with ESBL-PE status as the dependent variable. P values <0.05 were considered to be statistically significant.

### Ethical clearance

The study was approved by the Ministry of Health and the National Ethics Committee of Madagascar. Informed written consent was obtained from subjects and at least one parent of each child before enrollment.
